# Impaired skeletal muscle health in Parkinsonian syndromes: clinical implications, mechanisms and potential treatments

**DOI:** 10.1002/jcsm.13312

**Published:** 2023-08-13

**Authors:** Kate T. Murphy, Gordon S. Lynch

**Affiliations:** ^1^ Department of Anatomy and Physiology, Centre for Muscle Research The University of Melbourne Melbourne Australia

**Keywords:** Multiple system atrophy, Parkinson's disease, Progressive supranuclear palsy, Skeletal muscle, Wasting, Weakness

## Abstract

There is increasing evidence that neurodegenerative disorders including the Parkinsonian syndromes are associated with impaired skeletal muscle health, manifesting as wasting and weakness. Many of the movement problems, lack of muscle strength and reduction in quality of life that are characteristic of these syndromes can be attributed to impairments in skeletal muscle health, but this concept has been grossly understudied and represents an important area of unmet clinical need. This review describes the changes in skeletal muscle health in idiopathic Parkinson's disease and in two atypical Parkinsonian syndromes, the most aggressive synucleinopathy multiple system atrophy, and the tauopathy progressive supranuclear palsy. The pathogenesis of the skeletal muscle changes is described, including the contribution of impairments to the central and peripheral nervous system and intrinsic alterations. Pharmacological interventions targeting the underlying molecular mechanisms with therapeutic potential to improve skeletal muscle health in affected patients are also discussed. Although little is known about the mechanisms underlying these conditions, current evidence implicates multiple pathways and processes, highlighting the likely need for combination therapies to protect muscle health and emphasizing the merit of personalized interventions for patients with different physical capacities at different stages of their disease. As muscle fatigue is often experienced by patients prior to diagnosis, the identification and measurement of this symptom and related biomarkers to identify early signs of disease require careful interrogation, especially for multiple system atrophy and progressive supranuclear palsy where diagnosis is often made several years after onset of symptoms and only confirmed post‐mortem. We propose a multidisciplinary approach for early diagnosis and implementation of personalized interventions to preserve muscle health and improve quality of life for patients with typical and atypical Parkinsonian syndromes.

## Introduction

The Parkinsonian syndromes include idiopathic Parkinson's disease (PD) and the atypical syndromes, multiple system atrophy (MSA), progressive supranuclear palsy (PSP), corticobasal degeneration, and dementia with Lewy bodies, where patients exhibit some clinical features of PD with additional atypical symptoms.^1^ These conditions are associated with impaired mobility, including slowness, stiffness, balance and gait problems,^2^ and a loss of muscle strength which can lead to significant disability.^3^ Many of these symptoms can be attributed to impairments in skeletal muscle health.

Little is known about muscle health in Parkinsonian syndromes and whether the impairments are a consequence of denervation, disuse, malnutrition or intrinsic changes within skeletal muscle. Some studies have reviewed muscle fibre size and fibre composition in PD^4^ but have not interrogated the cellular and molecular mechanisms underlying these modifications. This is essential for devising strategies to preserve muscle function, promote mobility and improve quality of life for patients with Parkinsonian syndromes. Rehabilitation strategies have traditionally focused on central motor control,^4^ but combining these with ways to protect muscle health would likely elicit greater benefits than either approach alone.

This review is focused on PD and on MSA as the most aggressive synucleinopathy and PSP as a tauopathy. With no effective treatment currently available for these conditions, we identify potential mechanisms underlying each condition including roles for α‐synuclein (α‐syn) and tau in skeletal muscle, assess the efficacy of trials to preserve muscle health and discuss novel therapeutic targets deserving of further investigation.

## Methods

A comprehensive PubMed database search was performed from earliest entries to April 2023 using the terms ‘muscle wasting’, ‘skeletal muscle’, ‘sarcopenia’, ‘muscle atrophy’, ‘muscle health’ and ‘skeletal muscle health’ in combination with ‘Parkinson's disease’, ‘multiple system atrophy’, and ‘progressive supranuclear palsy’. Articles were further identified by searching for authors with direct expertise. No language, publication type or date restrictions applied to the searches.

Inclusion criteria included studies examining muscle parameters at a single time point, or changes over multiple time points, mechanisms, biomarkers, nutritional interventions, exercise and pharmacological inverventions.

Studies that included the same cohort as reported in other studies and those where full text was not available or were not published in English were excluded.

## The role of α‐synuclein and tau in skeletal muscle

### α‐Synuclein in skeletal muscle

Given the role of aberrant α‐synuclein (α‐syn) conformations and localization in the aetiology of PD and MSA, it is important to discuss the function of α‐syn in skeletal muscle. In a healthy muscle, α‐syn is localized at the postsynaptic region of the neuromuscular junction (NMJ)^5^ and important for nerve‐muscle communication, with whole body α‐syn knockout mice exhibiting impaired rotarod performance and decreased repetitive compound muscle action potentials during submaximal stimulation.^6^ The results implicated α‐syn in compartmentalization of the neurotransmitter acetylcholine (ACh) at the NMJ.^6^ An additional role for α‐syn in regulating glucose transport and insulin secretion is evident in mice, with α‐syn knockout causing glucose intolerance and insulin resistance^7^ but overexpression improving glucose tolerance and insulin sensitivity.^7^

Abnormal α‐syn localization may be associated with muscle pathology, with α‐syn present in ~60% of amyloid‐β (Aβ) positive vacuolated muscle fibres, and co‐localizing with Aβ in biopsies from patients with sporadic inclusion‐body myositis.^5^ Increased α‐syn immunoreactivity in necrotic muscle fibres, suggested a role in necrosis.^5^ Furthermore, overexpression of human α‐syn in transgenic mice reduced muscle mass and fibre size, increased fibrosis, and induced a fast‐to‐slow shift in muscle fibre composition.^5^ Muscles from transgenic mice also exhibited increased numbers of abnormal mitochondria in the intramuscular axons and NMJs, leading to inhibition of ACh release and fragmentation of ACh receptors, with increased reactive oxygen species (ROS) production and delayed regeneration after cardiotoxin injury.^5^ Thus, low levels of α‐syn expression and correct localization at the postsynaptic region of the NMJ is important for normal muscle function and glucose transport, but high levels and mislocalization of α‐syn can lead to muscle wasting and dysfunction. Increased α‐syn protein expression was detected in muscles from the methyl‐4‐phenyl‐1,2,3,6‐tetrahydropyridine (MPTP) mouse model of PD^8^ and treatment of C2C12 muscle cells with 1‐methyl‐4‐phenylpyridinium increased α‐syn protein expression.^9^ Furthermore, α‐syn expression was identified in extracellular medium from isolated skeletal muscles from aged rats.^10^ These findings indicate that skeletal muscle may be a source of extracellular α‐syn with the potential to cross the blood–brain barrier, but further studies are required to investigate the implications for PD and other neurodegenerative disorders.

### Tau in skeletal muscle

Tau is primarily localized in axons and important for microtubule stabilization and neurite growth.^11^ Under pathological conditions, however, tau becomes abnormally phosphorylated, conformationally altered, and aggregates into insoluble neurofibrillary tangles and in neuropil threads characteristic of the tauopathies, including Alzheimer's disease, corticobasal degeneration, Pick's disease, frontotemporal lobar degeneration and PSP.^12^ Tau is also expressed in non‐neuronal tissue including skeletal muscle^13^ despite fewer microtubules present in adult skeletal muscle [S1]. Tau deficient mice exhibit motor deficits and muscle weakness, with impaired performance in a wire‐hanging test.^14^ The PS19 transgenic mouse model of tauopathy that expresses a mutant form of human tau and exhibits hyperphoshorylated and insoluble mutant human tau protein [S2] had reduced grip strength and decreased mass and NMJ innervation of muscle, effects that were attenuated with forced expression of neuron‐specific calpastatin.^15^ Muscle fibre atrophy, reduced neuromuscular innervation and motor deficits were observed in transgenic Tau58/4 mice, which have a neuron‐specific expression of a mutant form of human tau.^16^ Future studies should employ muscle‐specific modulation of tau expression and phosphorylation status to better understand its role in skeletal muscle.

## Parkinson's disease

PD is characterized by damage to the dopaminergic neurons of the substantia nigra (SN) pars compacta (SNpc) and aggregation of misfolded α‐syn in intra‐cytoplasmic inclusions called Lewy bodies.^17^ PD affects ~0.3% of the general population and 1–3% over the age of 65,^18^ although these numbers are projected to double in the next two decades. Mean age of onset is 60 years and mean disease duration from diagnosis to death is 15 years, although patients can live for decades with PD.^18^ PD is more prevalent in men with males typically experiencing more severe symptoms [S3], including motor features (resting tremor, muscle rigidity, bradykinesia, postural and gait instability) and non‐motor features (dysautonomia, constipation, hyposmia, sleep disturbances and depression). Two‐thirds of patients are disabled within 5 years, and 80% are disabled after 10 years.^19^ Current treatment of PD is based on replacement of dopamine, although alternative approaches such as deep brain stimulation are suitable for later‐stage disease. Current treatments offer control of motor symptoms but do not halt progression of neurodegeneration, disease evolution or the increasing disability.

### Muscle health in Parkinson's disease

Weight loss (≥5% premorbid weight) occurs in 41–65% of PD patients^20^ [S4], with greater weight loss and lower body mass index (BMI) in women than men^21^ [S5]. Weight loss often begins years before diagnosis and increases as disease progresses^22^ [S6]. In PD patients, weight loss is associated with reduced quality of life, more severe parkinsonism, malnutrition, osteoporosis and fractures, dementia, worsening psychosis, dependency and death.^20^, ^22^, ^23^ Increased energy expenditure due to muscle rigidity and involuntary movements, lateral hypothalamic dysfunction, hyposmia, impaired hand‐mouth coordination, dysphagia, chewing difficulties, intestinal hypomobility and side effects of medications (including anorexia and nausea), all contribute to the weight loss.^24^ About half of PD patients experience fatigue, often manifesting prior to diagnosis and becoming one of the most debilitating symptoms.^25^ Despite a lack of investigation into the intrinsic changes within muscles of PD patients, the few studies in this area clearly identify impairments in muscle health (Table 1) that contribute to fatigue, weight loss and reduced quality of life.

**Table 1:** Skeletal muscle changes in patients with Parkinson's disease (PD)

Sample size, mean age (years), sex, mean years since diagnosis	Muscle, analysis type	Principal findings	Reference
*N* = 12, 67, M/F, 6	Vastus lateralis, muscle biopsy	Compared with age‐matched controls, PD had: 14% more type I fibres, 13‐fold larger type I fibre group size. Transcriptional profile assessed.	Lavin et al.^26^
*N* = 19, 67, M/F, unknown	Vastus lateralis, surface electromyography and muscle biopsy	Compared with age‐matched controls, PD had: increased percentage type I fibres, greater type I myofibre group size and motor unit over‐recruitment during submaximal sit‐to‐stand test, increased protein expression of MuSK.	Kelly et al.^27^
*N* = 27 (21 Dopa‐treated, 6 de novo), 66, M/F, 8.6	Biceps brachii or deltoid, muscle biopsy	Histochemistry: 10/17 PD patients had minor morphological abnormalities, including: ragged‐red fibres (*n* = 3); intermyofibrillar abnormalities (*n* = 7); and lobulated fibres (*n* = 6). Compared with controls, PD had: no change CS or SDH activity, mitochondrial complex I activity reduced by 71% Dopa‐treated, 68% de novo, complex III activity reduced by 35% Dopa‐treated.	Blin et al.^28^
*N* = 15, 66.5, M/F, 4.4	Vastus lateralis, muscle biopsy	Compared with age‐matched controls, PD had: increased percentage and CSA of type I fibres; no difference mitochondrial complex activity or CS activity; increased motor unit activation (+30%) during sit‐to‐stand test; no difference in leg strength, specific strength or power.	Kelly et al.^29^
*N* = 19, 58.2, M/F, 6.2	Vastus lateralis, muscle biopsy	Compared with age‐matched controls, PD had: mild to moderate neuropathic changes, mild fibre atrophy, dark staining for mitochondrial enzymes, increased CS activity (+36%), increase point mutated mtDNA (+12.6%), evidence of mild mitochondrial deficiency.	Winkler‐Stuck et al.^30^
*N* = 14, 60.6, M/F, 13.5	Paravertebral, muscle biopsy	Compared with controls, PD patients with camptocormia had: Increased variability in fibre size, hypertrophy of type I fibres, reduced number and size of type II fibres, increased % fibres with central nuclei, increased fibrosis, replacement of muscle cells with fat cells, decreased oxidative enzyme staining, myofibrillar disorganization, Z‐band streaming, electron‐dense patches. No signs of inflammation, mitochondriopathy or fibre type grouping.	Wrede et al.^31^
*N* = 8, 78.1, M/F, 16.6	Pharyngeal constrictor and cricopharyngeus, muscle sample post‐mortem	Compared with controls, PD patients had: Increased variability in fibre size, increased % fibres with central nuclei, more angular small fibres, fibre atrophy and fibre type grouping (greater atrophy in patients with dysphagia), fast‐to‐slow shift in fibre types, more N‐CAM‐positive fibres indicating denervation.	Mu et al.^32^

CSA, cross‐sectional area; CS, citrate synthase; SDH, succinate dehydrogenase.

Muscle biopsies from the vastus lateralis of PD patients revealed a unique pathological phenotype distinct from that of normal aging, with an increased grouping of slow, type I fibres associated with reduced quality of life.^26^, ^27^ This grouping was associated with an over‐recruitment of motor units during a sit‐to‐stand test, indicating reduced efficiency of motor unit recruitment.^27^, ^29^ Although sarcopenia (age‐related muscle atrophy) is associated with motor unit remodelling and a similar shift to more type I fibres, this was greater in PD.^26^, ^27^ Interestingly, type I muscle fibre size was greater in PD patients than age‐matched controls,^29^ which may be a mechanism to compensate for the preferential loss of type II motor units, and/or a consequence of increased type I motor unit recruitment.^29^

Camptocormia, the abnormal flexion of the thoracolumbar spine when standing or walking that disappears in the supine position, is experienced by ~7% of PD patients.^33^ Paravertebral muscles from patients with camptocormia have a loss of myofibril integrity, decreased oxidative enzyme activity, a shift towards more type I fibres and a reduced proportion (and atrophy) of type II fibres, as well as infiltration of connective tissue and fat.^31^ These effects contribute to the impaired ability to maintain an upright trunk.^31^

More than 50% of PD patients experience eating difficulties and masticatory dysfunction [S7] because of reduced efficiency of masseter and temporal muscles.^34^ PD patients recruit more muscle fibres during eating, contributing to the increased energy expenditure and leading to inadequate food intake, malnutrition, and weight loss.^34^ Facial tremors in PD may be due to reduced dopamine levels causing changes in mandible movement [S8], leading to dysphagia (impaired swallowing) experienced by 50–80% of PD patients, and consequent malnutrition and aspiration pneumonia, the leading cause of death in PD.^35^ Myopathic changes in the pharyngeal muscles controlling swallowing have been observed in PD, with atrophied and more centrally nucleated fibres, indicative of regeneration.^32^ These pharyngeal muscles also exhibit fibre grouping and a fast‐to‐slow shift in fibre type composition, consistent with impaired swallowing.^32^

Cardiac muscle is also affected in PD, with the changes reviewed extensively elsewhere [S10]. Briefly, orthostatic hypotension (OH) is experienced by a third of PD patients [S11] and associated with sympathetic denervation of the heart and impaired cardiac contractility and exercise performance.^36^ Cardiac sympathetic denervation also affects PD patients without orthostatic denervation, and progresses over time.^37^ PD patients had left ventricular hypertrophy, concentric remodelling, and diastolic dysfunction compared with age‐matched healthy controls.^38^ Furthermore, heart failure is 2.3‐times higher in PD patients compared with non‐PD controls [S12]. Some of the cardiovascular abnormalities may be side effects of PD treatment, with levodopa reducing blood pressure and aggravating OH [S10]. The uniqueness of the cardiovascular alterations in PD patients led to the term ‘the Parkinsonian Heart’^35^ and may be related to cardiac α‐syn aggregation; with α‐syn observed in epicardial nerve fascicles and myocardium in 60% and 40% PD patients but not in controls.^39^ Further research is required to confirm the mechanisms underlying cardiac dysfunction in PD.

### Genetics of Parkinson's disease

While most PD cases (85–90%) are idiopathic, 10–15% are familial, with genomic predispositions, aging, and cellular stressors, contributing to the risk.^40^ *SNCA*, the gene encoding for α‐syn protein, was the first gene associated with familial PD.^41^ Subsequently, *UCHL1*, *LRRK2*, *GIGYF2*, *HTRA2*, *EIF4G1*, *VPS35*, *DNAJC13*, *CHCHD2* and *PSAP* have been associated with autosomal dominant PD [S13]. Genes associated with autosomal recessive PD include *PRKN*, *PINK1*, *DJ‐1*, *TP13A*, *PLA2G6*, *FOBX7*, *DNAJC6*, *SYNJ* and *VPD13C* [S13]. The mutations identified in PD and the function of encoded proteins have been reviewed [S13‐S16]. The genetics of PD patients vary across ethnicities,^40^ but *PRKN* mutations are the most frequent cause of autosomal recessive PD and the most common genetic cause for early‐onset PD, while mutations in the *LRRK2* gene are most frequently the cause of late‐onset autosomal dominant PD [S14]. Whether muscle health impairments in PD patients are affected by the genetic mutation has not been investigated but could partly explain the diversity of PD symptoms and large inter‐individual variability. For example, *Drosophila* models of genetic PD caused by knockout or inactivation of either *PRKN* or *PINK1* exhibit impaired flight capacity and severe disruption to the integrity of flight muscles, which have irregular myofibrils and swollen mitochondria.^42^, ^43^ The findings suggest muscle health may be severely compromised in PD patients with *PRKN* or *PINK1* mutations, but this has yet to be confirmed, highlighting the need to examine muscle health in the context of the genetic mutation in patients with familial PD. It also supports precision medicine strategies^44^ where patients with genotypes associated with impaired muscle health could have customized treatments directed towards preserving mass and strength.

### Pathogenesis of impaired muscle health in Parkinson's disease

In addition to the potential role of increased skeletal muscle α‐syn expression, changes in the central and peripheral nervous system could contribute to impaired muscle health in PD. Compared with healthy, age‐matched controls, central activation during a quadriceps muscle activation test was lower in patients with advanced PD patients, but surprisingly, fatigue index was attenuated,^45^ a result the authors speculated could indicate insufficient central activation preventing muscle overload‐induced metabolic failure.^45^

PD patients exhibited a loss of type II motor units^46^ and an increase in type I motor units, but an over‐recruitment of type I motor units indicated impaired efficiency,^29^, ^47^ which could lead to impaired excitation‐contraction coupling, and reduced expression of Ca^2+^ regulatory proteins.^48^ Quadriceps femoris muscles from the MPTP mouse model of PD had reduced expression of sarcoplasmic reticulum (SR) Ca^2+^ release proteins, ryanodine receptors (RyR), calsequestrin 1 and triadin.^48^ Decreased expression of these proteins would impair SR Ca^2+^ release, thereby reducing force and speed of contraction and contributing to bradykinesia and tremor in PD patients. These findings are consistent with changes in Ca^2+^‐regulatory proteins in PD brains that contribute to neurodegeneration [S17]. Brains from PD patients had increased expression of the voltage‐gated Ca^2+^ channel subtype Ca_V_1 that preceded disease pathology, and greater relative expression of Ca_V_1.3 to Ca_V_1.2 subtypes, effects that would increase Ca^2+^ influx and enhance susceptibility of neurons to excitotoxicity or oxidative stress [S18]. Long‐term excessive cytosolic Ca^2+^ likely contributes to the ER, mitochondria, and lysosomal dysfunction in PD brains, as reviewed elsewhere [S19]. Strategies modulating Ca^2+^‐regulatory proteins to attenuate cellular Ca^2+^ overload have potential for reducing neurodegeneration and enhancing the force and speed of muscle contractions in PD patients.

Oxidative stress leading to mitochondrial dysfunction contributes to neurodegeneration in PD,^49^ but whether a similar mechanism exists in muscles of PD patients is inconclusive, with conflicting findings attributed to methodological differences and the characteristics of the patient population.^30^, ^50^, ^51^ The consensus is for mild and generalized muscle mitochondrial deficiency but a defect insufficient to contribute to the decrements in strength.^28^, ^30^, ^51^

**Figure 1:**
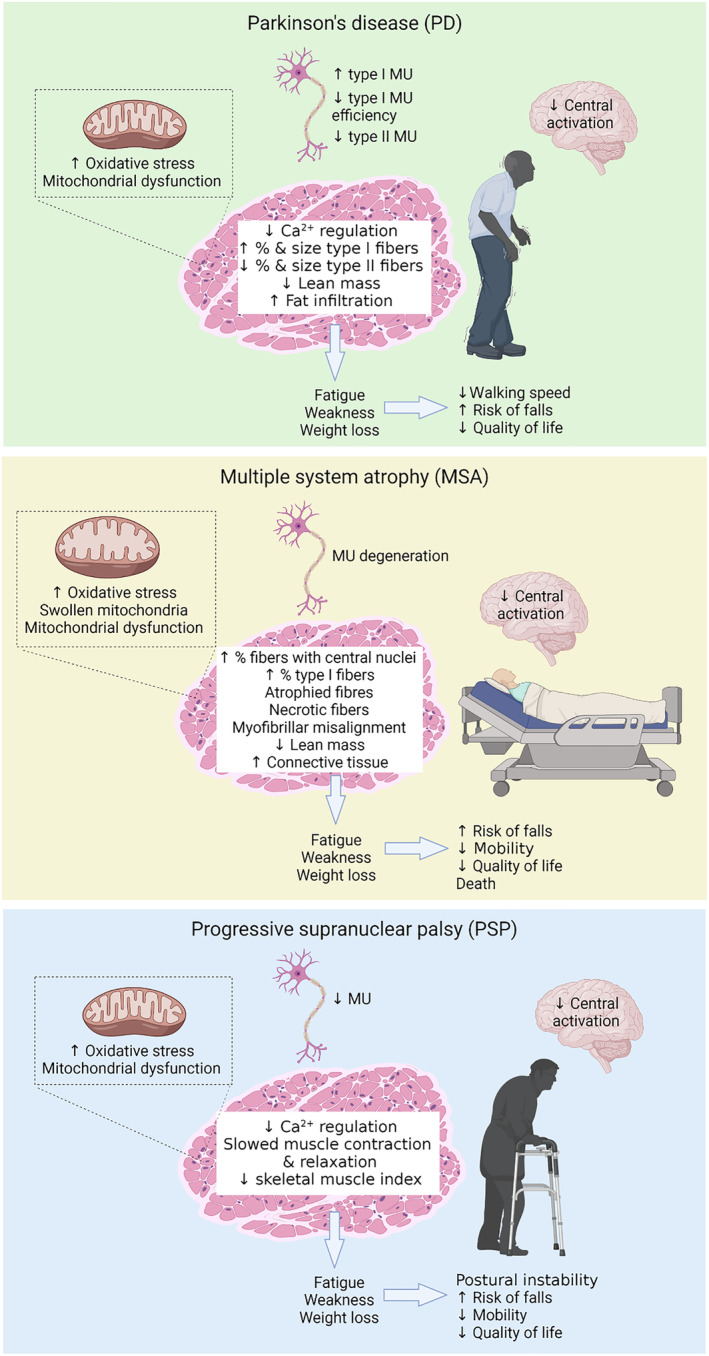
Summary of known mechanisms underlying changes in muscle health and the associated clinical outcomes in patients with Parkinson's disease, multiple system atrophy and progressive supranuclear palsy. MU, motor unit. Created with BioRender.com.

These defects in muscle health result from a combination of impairments in the central and peripheral nervous systems, and intrinsic changes within the muscle, including diminished regulation of intracellular Ca^2+^ and possibly increased oxidative stress (Figure 1).

### Interventions to improve muscle health in Parkinson's disease

#### Exercise

Exercise can improve PD symptoms and potential benefits have been reviewed [S20‐S22]. Sixteen weeks of thrice weekly resistance training in PD patients increased the cross‐sectional area of type II muscle fibres and improved both knee extension strength and quality of life.^52^ In the same study, transcript‐wide RNA‐sequencing of muscle biopsies revealed many genes significantly upregulated with resistance training are important for muscle remodelling, muscle and nerve development, inflammation and muscle metabolism.^53^ Many genes downregulated with resistance training are important for negatively regulating skeletal muscle growth, such as myostatin, a negative inhibitor of muscle size [S23], and for autophagy and slow muscle metabolism.^52^

Many of the well‐known neuroprotective effects of exercise in PD and other neurodegenerative diseases are attributed to crosstalk along the muscle‐brain axis.^54^ Skeletal muscle is an endocrine organ that produces, expresses, and secretes myokines,^55^ which are cytokines or other peptides produced during muscle contractions that exert paracrine, autocrine, and endocrine effects, including interleukin IL‐10, IL‐6, IL‐8, IL‐15, fibroblast growth factor 21, irisin, brain‐derived neurotrophic factor, cathepsin B, leukaemia inhibitory factor and Follisatin‐like‐1.^55^ Many of these myokines act directly on mitochondria to reduce oxidative stress and inflammation and enhance mitochondrial biogenesis. IL‐10 is a potent anti‐inflammatory cytokine that (in the brain) modulates astroglial activation and neuroinflammation [S24]. In inflammatory macrophages, IL‐10 regulates mitochondrial dynamics and bioenergetics towards an oxidative phenotype [S25]. Circulating IL‐6 levels can increase up to 100‐fold during exercise; the magnitude of change related to the type, duration, and intensity of exercise [S26]. IL‐6 enhances mitochondrial biogenesis in astrocytes under experimental septic conditions [S27] and stimulates production of IL‐10 and IL‐1 receptor antagonist (IL‐1ra), another anti‐inflammatory cytokine [S28]. However, while the transitory increase in IL‐6 with exercise induces anti‐inflammatory effects, sustained elevations contribute to the neuropathology of inflammatory diseases [S29]. Aerobic exercise is a potent stimulus for secretion of irisin, a soluble peptide with well‐known effects in adipose tissue where it contributes to the transition of white adipose to brown adipose [S30]. Irisin may also have neuroprotective effects in PD, with irisin treatment attenuating dopaminergic neuron loss and reducing α‐syn expression in mouse models of PD.^56^ Irisin triggers expression of brain‐derived neurotrophic factor [S31], which crosses the blood–brain barrier to enhance mitochondrial biogenesis in neurons and decrease dopaminergic loss in an animal model of PD [S32].

Myokines and other muscle‐derived molecules, such as mtDNA and microRNAs, are thought to be transported to the brain and other cells via exercise‐released extracellular vesicles [S33, S34]. Exercise may stimulate transfer of muscle mitochondria and mitochondrial DNA (mtDNA) via extracellular vesicles to repair damaged neuronal mitochondria and rescue respiration in the brain.^54^ Whether this contributes to the benefits of exercise for the PD brain, requires further investigation.

#### Nutritional interventions

Nutritional strategies with anti‐oxidative properties have therapeutic potential for maintaining muscle health in PD. Sulforaphane (SFN), a natural isothiocyanate in cruciferous vegetables (like broccoli, cabbage and cauliflower), upregulates erythroid 2‐related factor 2, which binds the antioxidant response element to drive expression of phase II antioxidant enzymes [S35] and maintain body redox homeostasis. SFN has relevance for conditions associated with oxidative stress and inflammation, and in mouse models of PD, SFN improved motor deficits, reduced oxidative stress, and protected dopaminergic neurons from neurodegeneration.^57^, ^58^ SFN attenuates atrophy of muscle cells in vitro [S36‐S38] and reduces muscle damage in mouse models of muscular dystrophy [S39‐S41], but its effect on muscle health in PD has not been investigated.

Other erythroid 2‐related factor 2 activators such as Tanshinone I, a lipophilic diterpenoid quinine in Danshen extract, and curcumin, a polyphenolic compound extracted from turmeric rhizome, improved motor function, reduced striatal oxidative stress, and protected against dopaminergic neuronal loss in the 6‐hydroxydopamine^59^ and MPTP mouse models of PD,^60^, ^61^ and attenuated atrophy and oxidative stress in models of muscle injury [S42, S43]. Whether similar protective effects are evident in muscles from PD patients has not been examined.

Other muscle‐targeted nutritional interventions include whey protein‐based formulas enriched with essential amino acids. One formula enriched with leucine and vitamin D combined with intensive rehabilitation in PD, MSA and PSP patients, improved walking distance and speed, and increased muscle mass more than rehabilitation alone.^62^ Based on the small sample size, the data should be interpreted with caution but highlight the need for larger studies that can discriminate between clinical entities.^62^

#### Pharmacological interventions

**Figure 2:**
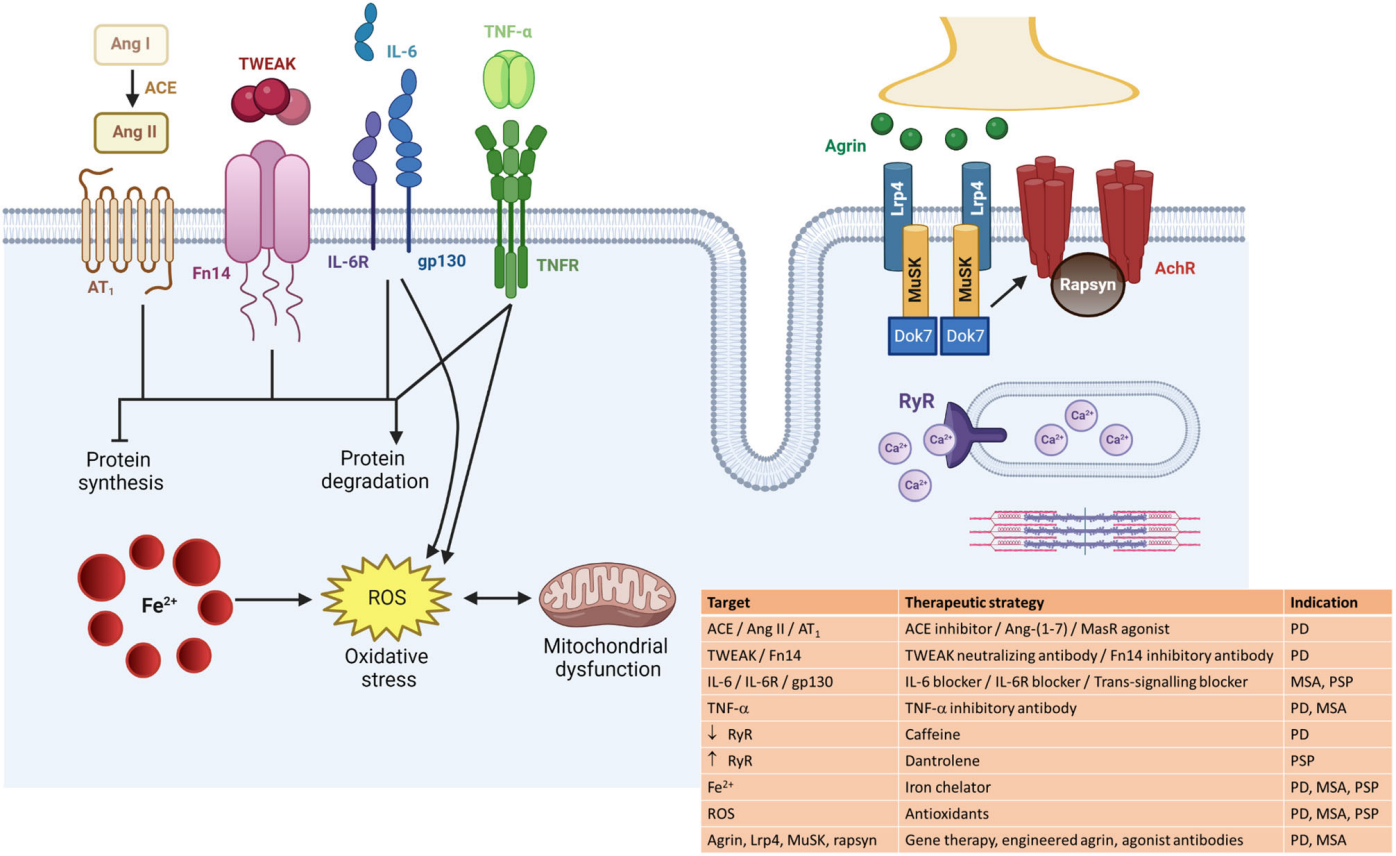
Potential targets and therapeutic strategies to improve skeletal muscle health in Parkinson's disease (PD), multiple system atrophy (MSA), and progressive supranuclear palsy (PSP). Ang I, angiotensin I; ACE, angiotensin converting enzyme; Ang II, angiotensin II; AT_1_, angiotensin II type 1 receptor; TWEAK, tumour necrosis factor‐like weak inducer of apoptosis; Fn14, fibroblast growth factor‐inducible 14; IL‐6, interleukin‐6; IL‐6R, interleukin‐6 receptor; gp130, glycoprotein 130 receptor; TNF‐α, tumour necrosis factor‐α; TNFR, tumour necrosis factor receptor; RyR, ryanodine receptor; Ca^2+^, calcium; Fe^2+^; iron; ROS, reactive oxygen species; Lrp4, lipoprotein receptor‐related protein 4; MuSK, muscle‐specific kinase; AChR, acetylcholine receptor. Created with BioRender.com.

No studies have examined the therapeutic potential of pharmacological interventions for muscle wasting and weakness in PD, but several show promise based on their ability to treat specific symptoms and attenuate muscle wasting in other conditions (Figure 2). This includes angiotensin converting enzyme (ACE) inhibitors, with their use in a cohort of 194 PD patients being associated with a reduced falls risk, independent of blood pressure.^63^ Reduced muscle strength is associated with an increased risk of falls in PD^64^ and ACE inhibitors can improve muscle function and fatigue resistance in mouse models of muscle wasting [S44, S45]. In contrast, stimulation of the “alternative” axis of the renin‐angiotensin system, involving ACE2, angiotensin‐(1‐7) (Ang‐(1‐7)) and the mitochondrial assembly receptor protected against cancer‐induced muscle wasting [S46] and improved motor performance and muscle co‐ordination in a 6‐hydroxydopamine rat model of PD.^65^ A combination of ACE inhibitors and activators of the ACE2/Ang‐(1–7)/mitochondrial assembly receptor axis represents a potential treatment for muscle wasting and weakness in PD.

Members of the tumour necrosis factor (TNF) superfamily of cytokines are linked to the pathogenesis of PD since TNF‐α levels were increased in PD brains [S47, S48] and ablation of TNF‐α protected against neurotoxicity in the MPTP mouse model of PD.^66^, ^67^ The more recently characterized TNF superfamily members, tumour necrosis factor‐like weak inducer of apoptosis (TWEAK) and its cognate receptor, fibroblast growth factor‐inducible 14 (Fn14), have been implicated in the aetiology of PD. Serum TWEAK levels were elevated in PD patients [S49] and treatment with a neutralizing TWEAK antibody attenuated dopaminergic cell death in the SNpc induced by sub‐acute MPTP injection.^68^ TWEAK/Fn14 contributes to muscle wasting associated with cancer [S50], aging [S51], denervation [S52], starvation [S53], and myotonic dystrophy [S54], but whether TWEAK and/or Fn14 levels are elevated in PD muscles and neutralizing antibodies can confer protection against PD‐associated muscle wasting is unknown and worthy of investigation.

Given the NMJ instability and evidence of muscle denervation in PD,^27^, ^29^ interventions targeting components of the NMJ, including agrin, lipoprotein receptor‐related protein 4, muscle‐specific kinase, and rapsyn, have potential to restore NMJ structure and function. Increasing expression of agrin, lipoprotein receptor‐related protein 4, muscle‐specific kinase, and rapsyn, improved NMJ formation and reduced denervation in mouse models of muscular dystrophy [S55‐S57], sarcopenia [S58, S59], and amyotrophic lateral sclerosis (ALS) [S60], but has yet to be investigated in PD.

Based on reduced expression of the SR Ca^2+^ release protein, the RyR, in muscles from MPTP mice,^48^ pharmacological activators such as caffeine have potential to improve force and contraction speed in PD.^69^

The redox active metal iron (Fe^2+^) binds α‐syn and promotes its aggregation and formation of Lewy bodies [S61, S62]. PD patients have increased regional iron levels in the SNpc [S63] and iron levels were elevated in the SN of MPTP‐treated mice [S64,^70^]. The iron chelator, deferiprone, reduced SN oxidative stress and improved motor function in MPTP mice.^71^ In the same study, deferiprone treatment decreased SN iron deposition and improved motor indicators of disease progression in patients with early‐stage PD.^71^ Peripheral infusion of ceruloplasmin, an endogenous ferroxidase involved in cellular iron efflux, reduced SN iron overload, and improved motor symptoms in MPTP‐treated mice.^72^ Similarly, the brain‐penetrant compound, ATH434 (formerly called PBT434), reduced iron accumulation, iron‐mediated redox activity, and neurotoxicity, and rescued motor performance in multiple rodent models of PD.^70^ Little is known about the role of iron in muscle wasting, especially in neurodegenerative disorders,^73^ but iron overload induced oxidative stress and reduced muscle mass, endurance, and strength [S65]. Sarcopenia in mice was characterized by increased muscle iron levels, lipid peroxidation, and fibre atrophy [S66], with changes in iron metabolism preceding symptoms in a rat model of ALS [S67]. Other studies report increased iron levels in severely affected diaphragm muscles of the *mdx* mouse model of Duchenne muscular dystrophy and treatment with the iron chelator, deferiprone, reduced fibrosis and oxidative stress in this muscle [S68]. A role for iron homeostasis in the regulation of muscle mitochondrial respiration and phenotype was implicated in a study that modelled mitochondrial dysfunction in PD by silencing the mitophagy gene, PTEN‐induced kinase 1 (Pink1), in *Drosophila*.^74^ Pink1 deficient *Drosophila* exhibited abnormal wing posture, reduced jumping activity, and thorax defects, abnormalities rescued by modulation in the flight muscles of the iron regulatory genes, *Drosophila* ZIP13 (dZIP13) and transferrin 1 (Tsf1), leading to increased mitochondrial iron content but no change in mitophagy.^74^ Whether a similar mechanism exists in muscles of PD patients should be investigated.

## Atypical parkinsonian syndromes

### Multiple system atrophy

MSA, a formerly known as Shy Drager syndrome, is a rare, rapidly progressing and fatal neurodegenerative disorder characterized by accumulation of α‐syn in oligodendrocytes and often in neurons, leading to demyelination and neurodegeneration.^53^ MSA is an orphan disease affecting 1.9–4.9 out of every 100 000 people^75^ [S69]. Mean disease onset is ~55 years of age and disease progression is rapid, with a mean survival of only 7–9 years after onset of symptoms^75^ [S69], including autonomic nervous system failure (manifesting as urogenital, gastrointestinal, and cardiovascular dysfunction leading to OH), motor, and non‐motor impairments.^76^ A poor initial response to dopamine replacement strategies differentiates MSA from PSP.^77^ MSA is typically divided into Parkinsonian (MSA‐P, with motor abnormalities including bradykinesia, rigidity and postural instability) and cerebellar categories (MSA‐C, including ataxia). Approximately 50% of MSA patients require a walking aid within 3 years after onset of motor symptoms, 60% need a wheelchair after 5 years, and most are bedridden within 6–8 years.^78^ Some symptoms can be treated with medications, but there are currently no treatments to slow disease progression, improve quality of life, or cure the condition. The development of disease‐modifying interventions is therefore an unmet clinical need in MSA.^76^

#### Muscle health in multiple system atrophy

Weight loss (≥10% premorbid weight) is experienced by the majority (~76%) of MSA patients but is less common among those with tube feeding (5–31%) compared to those without tube feeding (11–69%).^79^ The decrease in BMI of MSA‐P patients is associated with disease severity,^80^ although this relationship may be study‐specific since comparisons between independent, wheelchair‐bound, and bedridden MSA patients, found no difference in BMI despite caloric intake being reduced with disease severity.^81^ Fatigue is typically associated with weight loss in MSA and fatigue after exercise is one of the first symptoms reported by patients.^82^

One of the main causes of weight loss and fatigue in MSA is muscle wasting, which was first reported in 1983.^83^ Severe muscle wasting compromises survival and accounts for up to 45% of MSA deaths, with 30% attributed to respiratory (diaphragm) and/or cardiac muscle (heart) failure (cardiopulmonary arrest) and 15% attributed to being a ‘wasting syndrome’.^79^ Despite contributing to many clinical features of MSA, few studies have examined its skeletal muscle pathophysiology (Table 2).

**Table 2:** Skeletal muscle changes in patients with probable multiple system atrophy (MSA).

Sample size, mean age (years), sex, mean years since diagnosis	Muscle, analysis type	Principal findings	Reference
*N* = 1, 72, M, 6	Cervical paraspinal, muscle biopsy	Increase in connective tissue, regenerating and necrotic fibres, increased fibre size variation, increased percentage type I fibres, presence of internal nuclei, internal cores with reduced staining for oxidative enzymes, absence of inflammatory features.	van de Warrenburg et al.^84^
*N* = 40, 55.1, M/F, 5	Skeletal muscle electromyography	Abnormalities suggesting partial denervation found in 9 patients (22.5%) (fibrillations and fasciculations, *n* = 5; polyphasic compound muscle action potentials, *n* = 9; and large motor units, *n* = 9).	Pramstaller et al.^85^
*N* = 48, 60.6, M/F, 3.5	First interosseus dorsal muscle and tibialis anterior muscle, electromyography	Neurogenic type of abnormalities suggesting chronic reinnervation (high motor unit potential amplitude and increased size index) were observed in 44.7% of examined muscles. No evidence of acute denervation (fibrillations and positive sharp waves).	Gawel et al.^86^
*N* = 5, 66.6, unknown, 5	Biceps brachii or deltoid, muscle biopsy	Histochemistry: 1/5 MSA patients had intermyofibrillary abnormalities and vacuolized muscular cells. Compared with age‐matched controls, MSA had: No change SDH or CS activity, 75% lower mitochondrial complex I activity.	Blin et al.^28^
*N* = 27, 59.9, M/F, 3.6	Respiratory, maximal respiratory pressures	Compared with age‐matched controls, MSA had: 22% lower maximum inspiration pressure and 27% lower maximum expiration pressure.	Wang et al.^87^
*N* = 1, 68, M, 8	Posterior cricoarytenoid laryngeal, total laryngectomy	Abnormal swollen mitochondria and myofilaments, distorted Z lines, muscle fibre degeneration and replacement with connective tissue. Ultrastructural changes in NMJs: abnormal synaptic contacts, flattened primary synaptic clefts, replacement of nerve terminals by Schwann cells.	Yoshihara et al.^88^
*N* = 1, 75, F, 2	Oesophagus and detrusor, post‐mortem histopathology	Upper cervical oesophagus: muscle fibre atrophy, eosinophilic infiltration, fibrosis, necrosis. Urinary bladder: muscle fibre degeneration and eosinophilic infiltration.	Song et al.^89^

CS, citrate synthase; SDH, succinate dehydrogenase.

Wasting of limb and respiratory muscles robs patients of their strength and capacity to perform daily tasks and live independently and leads to motor impairments closely associated with poor quality of life scores,^90^ including ataxia, gait, and balance issues, limb contractures, and compromised respiratory muscle strength with abnormal pulmonary function.^87^ Histochemical analysis of biceps brachii and deltoid muscles from five patients with probable MSA revealed a 75% lower mitochondrial complex I activity and the presence of vacuolized cells compared with controls.^28^ There was no difference between groups in muscle citrate synthase or succinate dehydrogenase (SDH) activity, or in complex II, III and IV activity.^28^ A limitation was that the controls were younger (mean age, 58.4 years) than MSA patients (mean age, 66.6 years), and so larger studies using age‐matched controls are needed.

MSA patients exhibit pathology in the neck muscles, which contributes to the disproportionate antecollis observed in this population. A biopsy of the cervical paraspinal muscles in one PD and one MSA patient revealed a common pathology, including infiltration of connective tissue, greater variation in muscle fibre size, and presence of central nuclei,^84^ except the biopsy from the MSA patient showed necrotic fibres, an increased proportion of type I fibres, and abnormal fibres with reduced core oxidative enzyme reactivity.^84^ The worsened pathology could contribute to the earlier onset of disproportionate antecollis in MSA compared with PD patients, but comprehensive studies are required for confirmation.

Dysfunction of the laryngeal muscles contributes to dysphagia and vocal impairments in MSA and in a 68‐year‐old MSA patient undergoing total laryngectomy, ultrastructural analysis of the posterior cricoarytenoid laryngeal muscle, the muscle that opens the glottis, revealed significant changes at the NMJ, characterized by motor nerve degeneration in both the pre‐ and post‐synaptic regions.^88^ These changes were associated with muscle fibre atrophy, Z‐line disorganization, swollen mitochondria, and connective tissue infiltration.^88^

Urinary incontinence is one of the first symptoms of MSA and post‐mortem examination of the detrusor muscle in a 75‐year‐old female patient revealed muscle fibre degeneration and eosinophilic infiltration.^89^ Other notable muscle groups likely affected in MSA but not yet studied extensively, include the masseter muscles, which contribute to the loss of masticatory function and eating difficulties,^91^ the gastrointestinal tract, that likely leads to the colonic motility impairments in MSA,^92^ and the extraocular muscles, which could contribute to visual disturbances.^93^

Cardiac failure has been reported as the primary cause of sudden death in MSA patients (33%),^9^ but the underlying mechanisms remain unknown. Unlike PD, sympathetic denervation is rare in MSA,^39^ but OH occurs frequently and more so in MSA‐P than MSA‐C.^94^ The severity of OH, increased variability in daytime systolic blood pressure and OH drug treatment were all independently correlated with mortality in MSA patients^94^ and raise concerns about OH drug treatment in this population. Although α‐syn aggregation has been observed in epicardial nerve fascicles and myocardium of MSA patients^39^ [S70], its association with cardiac function requires further investigation.

#### Pathogenesis of muscle health impairments in multiple system atrophy

The pathogenesis of muscle wasting in MSA is unknown but likely involves both central and peripheral mechanisms. Altered α‐syn expression and/or conformation may also contribute to the muscle dysfunction in MSA patients but has yet to be studied. Central effects include pro‐inflammatory cytokines such as TNF‐α and IL‐6 released into the systemic circulation by microglia activation [S71] and their endocrine effects on skeletal muscle. Serum TNF‐α and IL‐6 levels were elevated 16% and 7%, respectively, in MSA patients,^95^ increasing ubiquitin ligase‐mediated muscle protein degradation [S72], and TNF‐α directly supressing force production [S73]. Whether cytokine levels are increased in MSA muscle has not been examined.

Microglia activation and neuroinflammation can lead to release of ROS [S71], and in transgenic mice, the MSA pathology is modelled by overexpressing oligodenroglial α‐syn under conditions of oxidative stress caused by 3‐nitropropionic acid (3‐NP).^96^ Increased ROS induces oxidative damage within skeletal muscles [S74] and mitochondrial dysfunction has been demonstrated in MSA muscle.^28^, ^84^ Disuse increases muscle production of pro‐inflammatory cytokines and ROS [S73], and so prolonged inactivity in (especially bedridden) MSA patients would exacerbate these deleterious effects.

Peripheral neuron degeneration‐mediated denervation is another possible mechanism underlying muscle wasting in MSA, and Schwann cell α‐syn deposits have been observed in muscles from MSA patients^97^ and in the PLP‐α‐syn mouse model of MSA.^98^ However, peripheral neuron degeneration and partial muscle denervation have been found only in ~20–25% of MSA patients^85^, ^86^ and PLP‐α‐syn mice do not exhibit impairments in the peripheral nervous system.^98^ Reinnervation of motor neurons was found in ~45% MSA patients^86^ and an increased proportion of type I fibres^84^ suggests MSA may be associated with a specific denervation of fast myofibres and a subsequent reinnervation that remodels them into slow myofibres. Clearly, multiple mechanisms underpin the muscle wasting in MSA, including central and local production of pro‐inflammatory cytokines and ROS, and peripheral neuron degeneration‐mediated denervation (Figure 1), but focused studies are needed to understand the mechanisms underlying the pathological progression of MSA.

#### Interventions to improve muscle health in multiple system atrophy

##### Exercise

No study to date has tested whether physical exercise can attenuate muscle wasting and weakness in MSA. However, two studies have reported positive effects of resistance training on functional outcomes in MSA patients.^99^, ^100^ Although physical activity may be recommended in the early stages of MSA, it is not a viable option at the more advanced stages, and devising and testing alternative strategies, including functional electrical stimulation of peripheral nerves, and pharmacological interventions targeting underlying molecular mechanisms, are important and could help address a significant unmet clinical need.

##### Pharmacological interventions

No published studies have examined the therapeutic potential of pharmacological interventions to improve muscle pathology in MSA, but anti‐inflammatory and anti‐oxidative interventions have promise (Figure 2). Lenalidomide, a small thalidomide derivative that inhibits TNF‐α production, reduced α‐syn accumulation, astrogliosis and microgliosis, in MSA mice, but greater effects were found in combination with a single‐chain antibody targeting α‐syn modified for improved central nervous system penetration (CD5‐D5).^101^ Inhibition of myeloperoxidase (MPO), a key enzyme involved in ROS production, attenuated microglial activation, protected neurons, reduced α‐syn aggregation, and improved motor function in the PLP‐α‐syn mouse model of MSA.^102^ Whether these interventions improve muscle pathology and functional capacity is unknown.

Like PD, MSA patients have increased iron levels in the SNpc and the striatum [S63] and a case study observed iron deposition in the putamen of an MSA‐P patient preceded onset of clinical symptoms by 2 years [S75]. The PLP‐α‐syn mouse model of MSA exhibited an age‐ and region‐dependent increase in brain iron levels and treatment with deferiprone, ceruloplasmin, or ATH434 improved neuronal survival and motor performance and reduced α‐syn accumulation in the SNpc.^103^, ^104^ Whether muscle iron levels are increased in MSA patients or whether improvements in motor performance with iron‐modulating compounds are associated with reduced muscle iron are unknown and deserving of investigation.

### Progressive supranuclear palsy

PSP was first described in 1964^105^ and is the most common form of atypical Parkinsonism. PSP is considered a tauopathy, characterized by abnormal intracerebral aggregation of microtubule‐associated tau protein, the appearance of ‘tufted astrocytes’ and neurodegeneration of the SN, subthalamic nucleus, and midbrain.^105^ The typical symptom of PSP is the supranuclear palsy of vertical gaze, although this may not develop for 3–4 years after disease onset. Other symptoms include postural instability and balance issues with recurrent falls, oculomotor dysfunction, parkinsonism, and neuropsychiatric symptoms [S76]. Based on the predominant clinical features, there are several subtypes of PSP including PSP with parkinsonism (PSP‐P), PSP with progressive gait freezing (PSP‐PGF), and PSP with Richardson's syndrome (PSP‐RS) [S77]. The estimated incidence of PSP in the population aged 50 years and older was 5.0 per 100 000 person‐years, with greater incidence in men (7.6 per 100 000) than women (3.0 per 100 000) [S78]. PSP is typically diagnosed between 50 and 70 years of age with median survival time from symptom onset of 5.5–6 years [S76, S78]. Most patients require a non‐wheelchair walking aid within 1.5–2 years of disease onset, and a wheelchair within 3.5–5 years [S79]. Current therapies are symptomatic, and effective disease‐modifying treatments remain elusive. Levodopa can improve bradykinesia and rigidity in approximately one‐third of patients, but the benefits are generally transient, and prolonged improvement is considered among the exclusionary criteria for diagnosis of PSP.

#### Muscle health in progressive supranuclear palsy

Weight loss occurs earlier in the progression of PSP compared with PD.^106^ Fatigue was reported by ~39% PSP patients^107^ and was experienced by 10% patients prior to diagnosis indicating it may be an early sign of PSP [S80].

Few studies have examined muscle strength and size in PSP (Table 3), but during a grip strength task, PSP patients were slower at contracting and relaxing their muscles, produced longer pulse durations and were weaker than younger, healthy controls.^108^ They also produced additional force pulses due to cognitive impairment and/or diminished motor inhibition.^108^ A limitation was that the PSP group was older (mean age, 72.6 years) than the controls (mean age, 61.2 years), making it difficult to discern whether these differences were due to normal aging or disease. PSP patients had decreased skeletal muscle index compared with age‐matched controls^109^ but was correlated with age in females, suggesting that wasting may have been a consequence of normal aging in females, whereas in males it was likely induced by disease.^109^

**Table 3:** Skeletal muscle changes in patients with probable progressive supranuclear palsy (PSP)

Sample size, mean age (years), sex, mean years since diagnosis	Muscle, analysis type	Principal findings	Reference
*N* = 5, 69, M/F, 6.4	Gastrocnemius, phosphorous magnetic resonance spectroscopy	Compared with age‐matched controls, PSP had: Increased resting [P_i_] (+49%), no difference PCr, slowed rate of PCr post‐exercise.	Martinelli et al.^116^
*N* = 6, 72.5, M/F, 5.8	Biceps, muscle biopsy – mitochondria isolated	Compared with age‐matched controls, PSP had: No change CS activity; decreased rate of ATP production through complex I, III and IV (−29%), complex IV (−35%) but not through complex II, III and IV (*P* = 0.058). Decrease in mitochondrial respiratory chain activity in 4/6 patients. No difference muscle fibre size or fibre architecture.	Di Monte et al.^115^
*N* = 39, 73.8, M/F, 4.6	Body composition	Compared with age‐matched controls, PSP had: Reduced total SMI (−20% females, −8% males), reduced leg SMI (−25% females, −10% males), no change arm SMI, increased % patients with low SMI (+53% females, +41% males), decreased basal metabolism in females (−13%), increased frequency of obesity in females (+40%).	Takamatsuet al.^109^
*N* = 6, 68.5, M/F, unknown	Quadriceps, ^99m^Tc‐sestamibi SPECT analysis of muscle mitochondrial function	Compared with controls (mean age, 43.6 years), PSP had: Reduced radionucleotide uptake at 10 min (−45%) and 4 h (−46%) after ^99m^Tc‐sestamibi injection.	Chang et al.^114^
*N* = 24, 67.5, M/F, 3.5	First interosseus dorsal and tibial anterior, surface EMG	No signs of acute denervation, evidence of chronic reinnervation (high MUP amplitude, increased SI) in 42% PSP muscles.	Gawel et al.^113^
*N* = 12, 62.3, M/F, 5.9	Biceps, thenar and tibialis anterior,	Compared with age‐matched controls, PSP had: Central motor conduction abnormalities found in 42% patients, with long illness (>4 years) and increased appearance of pyramidal signs.	Abbruzzese et al.^112^

[P_i_], inorganic phosphate concentration; ^99m^Tc‐sestamibi, technetium‐99m methoxyisobutyl isonitrile; CS, citrate synthase; MUP, motor unit potential; PCr, phosphocreatine; SI, size index; SMI, skeletal muscle index; SPECT, single photon emission computed tomography; TMPD, *N*,*N*,*N*′,*N*′‐tetramethyl‐*p*‐phenylenediarnin.

Compared to MSA and PD, cardiac muscle appears relatively spared in PSP. Some studies have reported OH and impaired heart rate response to orthostatic stress^110^ while others have found little or no evidence of autonomic dysfunction in PSP^111^ [S81]. The sympathetic nerves were well preserved in PSP [S82] but whether α‐syn is expressed in these nerves or myocardium in PSP patients has not been investigated.

#### Pathogenesis of muscle health impairments in progressive supranuclear palsy

Like PD and MSA, impairments in the central and/or peripheral nervous system and intrinsic changes within the muscle may contribute to the decrements in muscle health in PSP (Figure 1). As discussed earlier, altered muscle expression or phosphorylation of tau might contribute to the muscle health impairments in PSP but has yet to be investigated. Central motor conduction was impaired in ~40% PSP patients^112^ and surface EMG recordings found abnormalities indicating loss of motor neurons in nearly half of the PSP patients examined.^113^ Muscles from PSP patients had evidence of chronic reinnervation without signs of acute denervation, suggesting a very slow disease progression.^113^

There is increasing evidence that mitochondrial impairments may contribute to oxidative stress and the pathogenesis of PSP [S83], including decreased alpha‐ketoglutarate dehydrogenase complex and aconitase [S84, S85] activities, in the superior frontal cortex and cerebella of PSP patients compared with age‐matched controls. However, glutamate dehydrogenase and complex I and IV activities were not altered in PSP [S84, S85], suggesting that the decline in ketoglutarate dehydrogenase complex and aconitase does not simply reflect a reduction in mitochondria number. More recent studies demonstrated reduced muscle mitochondrial function in PSP patients compared with younger, control subjects,^114^ but effects were not significantly correlated with age. Defects in mitochondrial oxidative phosphorylation were also detected in muscles from PSP patients although it was unclear whether these were attributed to disuse or the disease.^115^ Increased resting inorganic phosphate concentration and a slowed post‐exercise decrease in phosphocreatine in gastrocnemius muscles from PSP patients was further evidence of mitochondrial defects.^116^

#### Interventions to improve muscle health in progressive supranuclear palsy

##### Exercise

The benefits of exercise and structured physical therapy for PSP have been reviewed [S86]. Although some studies have reported benefits of weight‐supported treadmill training, music‐cued movement rehabilitation, and robotic gait training for improving walking speed in early onset PSP, many of these studies have been underpowered and inadequately reported key aspects of the exercise protocols and evaluation criteria [S87]. It remains inconclusive whether exercise can benefit patients with advanced PSP.

##### Pharmacological interventions

There have been no published studies investigating pharmacological interventions for preserving muscle health in PSP. Anti‐oxidative strategies should be tested for their ability to attenuate oxidative stress and improve muscle function. As in PD and MSA, excessive iron accumulation occurs in brains and motor nerves of PSP patients [S88, S89]. Therefore, iron chelators or other iron‐modifying compounds may have similar potential for protecting muscles in PSP.

Serum IL‐6 levels were correlated with disease severity in PSP^117^ and cerebrospinal fluid levels were higher in PSP than PD patients.^118^ As IL‐6 has been linked with the pathophysiology of tauopathies such as Alzheimer's disease [S90], anti‐IL‐6 strategies may prove beneficial for PSP.

The ryanodine receptor antagonist, dantrolene, reduced the frequency and severity of muscle spasms in a 45‐year‐old male PSP patient.^119^ Dantrolene inhibits SR Ca^2+^ release and so its efficacy for reducing muscle spasms indicates that dysregulated Ca^2+^ handling and a subsequent increase in intracellular [Ca^2+^] likely contribute to the muscle pathology in PSP.

## Conclusion

There is growing evidence that impairments in skeletal muscle health play an important role in the progression of PD, MSA and PSP and contribute to some of the most debilitating symptoms. Little is known about the mechanisms underlying these conditions, but multiple pathways and processes are implicated (Figure 2), indicating that combination therapies might best protect muscle health and underscoring the need for personalized interventions for patients with differing physical capacities. Investigations of muscle health in typical and atypical Parkinsonian syndromes remains understudied and represents an important area of unmet clinical need, especially to understand disease mechanisms and identify interventions to improve quality of life.

## Conflict of interest

Kate T. Murphy and Gordon S. Lynch declare that they have no conflicts of interest.

## Supporting information


Supporting Information S1
