# Supplementary material for: Impaired skeletal muscle health in Parkinsonian syndromes: clinical implications, mechanisms and potential treatments
Source: J Cachexia Sarcopenia Muscle. 2023 Aug 13;14(5):1987–2002. doi: 10.1002/jcsm.13312 (PMC10570091; doi:10.1002/jcsm.13312)
Supplement: Supplementary file 1 — Supporting Information S1 [file JCSM-14-1987-s001.docx]

**Additional References**

S1. Kano Y, Fujimaki N, Ishikawa H. The distribution and arrangement of microtubules in mammalian skeletal muscle fibers. Cell Struct Funct. 1991;16:251-61.

S2. Yoshiyama Y, Higuchi M, Zhang B, Huang SM, Iwata N, Saido TC, et al. Synapse loss and microglial activation precede tangles in a P301S tauopathy mouse model. Neuron. 2007;53:337-51.

S3. Haaxma CA, Bloem BR, Borm GF, Oyen WJ, Leenders KL, Eshuis S, et al. Gender differences in Parkinson's disease. J Neurol Neurosurg Psychiatry. 2007;78:819-24.

S4. Abbott RA, Cox M, Markus H, Tomkins A. Diet, body size and micronutrient status in Parkinson's disease. Eur J Clin Nutr. 1992;46:879-84.

S5. Durrieu G, ME LL, Rascol O, Senard JM, Rascol A, Montastruc JL. Parkinson's disease and weight loss: a study with anthropometric and nutritional assessment. Clin Auton Res. 1992;2:153-7.

S6 Chen H, Zhang SM, Hernan MA, Willett WC, Ascherio A. Weight loss in Parkinson's disease. Ann Neurol. 2003;53:676-9.

S7. Tomic S, Rajkovaca I, Pekic V, Salha T, Misevic S. Impact of autonomic dysfunctions on the quality of life in Parkinson's disease patients. Acta Neurol Belg. 2017;117:207-11.

S8. Robertson LT, Hammerstad JP. Jaw movement dysfunction related to Parkinson's disease and partially modified by levodopa. J Neurol Neurosurg Psychiatry. 1996;60:41-50.

S9. Johnston BT, Li Q, Castell JA, Castell DO. Swallowing and esophageal function in Parkinson's disease. Am J Gastroenterol. 1995;90:1741-6.

S10. Cuenca-Bermejo L, Almela P, Navarro-Zaragoza J, Fernández Villalba E, González-Cuello AM, Laorden ML, et al. Cardiac Changes in Parkinson's Disease: Lessons from Clinical and Experimental Evidence. Int J Mol Sci. 2021;22:13488.

S11. Senard JM, Rai S, Lapeyre-Mestre M, Brefel C, Rascol O, Rascol A, et al. Prevalence of orthostatic hypotension in Parkinson's disease. J Neurol Neurosurg Psychiatry. 1997;63:584-9.

S12. Zesiewicz TA, Strom JA, Borenstein AR, Hauser RA, Cimino CR, Fontanet HL, et al. Heart failure in Parkinson's disease: analysis of the United States medicare current beneficiary survey. Parkinsonism Relat Disord. 2004;10:417-20.

S13. Castillo-Rangel C, Marin G, Hernandez-Contreras KA, Vichi-Ramirez MM, Zarate-Calderon C, Torres-Pineda O, et al. Neuroinflammation in Parkinson's Disease: From Gene to Clinic: A Systematic Review. Int J Mol Sci. 2023;24:5792.

S14. Klein C, Westenberger A. Genetics of Parkinson's disease. Cold Spring Harb Perspect Med. 2012;2:a008888.

S15. Schiesling C, Kieper N, Seidel K, Kruger R. Review: Familial Parkinson's disease--genetics, clinical phenotype and neuropathology in relation to the common sporadic form of the disease. Neuropathol Appl Neurobiol. 2008;34:255-71.

S16. Selvaraj S, Piramanayagam S. Impact of gene mutation in the development of Parkinson's disease. Genes Dis. 2019;6:120-8.

S17. Schapira AH. Calcium dysregulation in Parkinson's disease. Brain. 2013;136:2015-6.

S18. Hurley MJ, Brandon B, Gentleman SM, Dexter DT. Parkinson's disease is associated with altered expression of Ca_V_1 channels and calcium-binding proteins. Brain. 2013;136:2077-97.

S19. Xu J, Minobe E, Kameyama M. Ca^2+^ Dyshomeostasis Links Risk Factors to Neurodegeneration in Parkinson's Disease. Front Cell Neurosci. 2022;16:867385.

S20. Feng YS, Yang SD, Tan ZX, Wang MM, Xing Y, Dong F, et al. The benefits and mechanisms of exercise training for Parkinson's disease. Life Sci. 2020;245:117345.

S21. Ellis T, Rochester L. Mobilizing Parkinson's Disease: The Future of Exercise. J Parkinsons Dis. 2018;8:S95-S100.

S22. Lamotte G, Rafferty MR, Prodoehl J, Kohrt WM, Comella CL, Simuni T, et al. Effects of endurance exercise training on the motor and non-motor features of Parkinson's disease: a review. J Parkinsons Dis. 2015;5:21-41.

S23. McPherron AC, Lawler AM, Lee SJ. Regulation of skeletal muscle mass in mice by a new TGF-β superfamily member. Nature. 1997;387:83-90.

S24. Porro C, Cianciulli A, Panaro MA. The Regulatory Role of IL-10 in Neurodegenerative Diseases. Biomolecules. 2020;10:1017.

S25. Dowling JK, Afzal R, Gearing LJ, Cervantes-Silva MP, Annett S, Davis GM, et al. Mitochondrial arginase-2 is essential for IL-10 metabolic reprogramming of inflammatory macrophages. Nat Commun. 2021;12:1460.

S26. Pedersen BK. Exercise-induced myokines and their role in chronic diseases. Brain Behav Immun. 2011;25:811-6.

S27. Chen XL, Wang Y, Peng WW, Zheng YJ, Zhang TN, Wang PJ, et al. Effects of interleukin-6 and IL-6/AMPK signaling pathway on mitochondrial biogenesis and astrocytes viability under experimental septic condition. Int Immunopharmacol. 2018;59:287-94.

S28. Steensberg A, Fischer CP, Keller C, Moller K, Pedersen BK. IL-6 enhances plasma IL-1ra, IL-10, and cortisol in humans. Am J Physiol Endocrinol Metab. 2003;285:E433-7.

S29. Muñoz-Canoves P, Scheele C, Pedersen BK, Serrano AL. Interleukin-6 myokine signaling in skeletal muscle: a double-edged sword? FEBS J. 2013;280:4131-48.

S30. Bostrom P, Wu J, Jedrychowski MP, Korde A, Ye L, Lo JC, et al. A PGC1-alpha-dependent myokine that drives brown-fat-like development of white fat and thermogenesis. Nature. 2012;481:463-8.

S31. Wrann CD, White JP, Salogiannnis J, Laznik-Bogoslavski D, Wu J, Ma D, et al. Exercise induces hippocampal BDNF through a PGC-1α/FNDC5 pathway. Cell Metab. 2013;18:649-59.

S32. Real CC, Ferreira AF, Chaves-Kirsten GP, Torrao AS, Pires RS, Britto LR. BDNF receptor blockade hinders the beneficial effects of exercise in a rat model of Parkinson's disease. Neuroscience. 2013;237:118-29.

S33. Trovato E, Di Felice V, Barone R. Extracellular Vesicles: Delivery Vehicles of Myokines. Front Physiol. 2019;10:522.

S34. Memme JM, Erlich AT, Phukan G, Hood DA. Exercise and mitochondrial health. J Physiol. 2021;599:803-17.

S35. Huang Y, Li W, Su ZY, Kong AN. The complexity of the Nrf2 pathway: beyond the antioxidant response. J Nutr Biochem. 2015;26:1401-13.

S36. Li W, Trieu J, Blazev R, Parker BL, Murphy KT, Swiderski K, et al. Sulforaphane attenuates cancer cell-induced atrophy of C2C12 myotubes. Am J Physiol Cell Physiol. 2023;324:C205-C21.

S37. Moon JY, Kim DJ, Kim HS. Sulforaphane ameliorates serum starvation-induced muscle atrophy via activation of the Nrf2 pathway in cultured C2C12 cells. Cell Biol Int. 2020;44:1831-9.

S38. Son YH, Jang EJ, Kim YW, Lee JH. Sulforaphane prevents dexamethasone-induced muscle atrophy via regulation of the Akt/Foxo1 axis in C2C12 myotubes. Biomed Pharmacother. 2017;95:1486-92.

S39. Sun C, Li S, Li D. Sulforaphane mitigates muscle fibrosis in *mdx* mice via Nrf2-mediated inhibition of TGF-beta/Smad signaling. J Appl Physiol (1985). 2016;120:377-90.

S40. Sun C, Yang C, Xue R, Li S, Zhang T, Pan L, et al. Sulforaphane alleviates muscular dystrophy in *mdx* mice by activation of Nrf2. J Appl Physiol (1985). 2015;118:224-37.

S41. Sun CC, Li SJ, Yang CL, Xue RL, Xi YY, Wang L, et al. Sulforaphane Attenuates Muscle Inflammation in Dystrophin-deficient mdx Mice via NF-E2-related Factor 2 (Nrf2)-mediated Inhibition of NF-kappaB Signaling Pathway. J Biol Chem. 2015;290:17784-95.

S42. Wang J, Cai J, Wang X, Zhu G, Feng Y, Chen H, et al. An injectable liposome for sustained release of tanshinone IIA to the treatment of acute blunt muscle injury by augmenting autophagy and alleviating oxidative stress. Am J Transl Res. 2020;12:4189-203.

S43. Vargas-Mendoza N, Madrigal-Santillan E, Alvarez-Gonzalez I, Madrigal-Bujaidar E, Anguiano-Robledo L, Aguilar-Faisal JL, et al. Phytochemicals in Skeletal Muscle Health: Effects of Curcumin (from Curcuma longa Linn) and Sulforaphane (from Brassicaceae) on Muscle Function, Recovery and Therapy of Muscle Atrophy. Plants (Basel). 2022;11:2517.

S44. Murphy KT, Chee A, Trieu J, Naim T, Lynch GS. Inhibition of the renin-angiotensin system improves physiological outcomes in mice with mild or severe cancer cachexia. Int J Cancer. 2013;133:1234-46.

S45. Burks TN, Andres-Mateos E, Marx R, Mejias R, Van Erp C, Simmers JL, et al. Losartan restores skeletal muscle remodeling and protects against disuse atrophy in sarcopenia. Sci Transl Med. 2011;3:82ra37.

S46. Murphy KT, Hossain MI, Swiderski K, Chee A, Naim T, Trieu J, et al. Mas receptor activation slows tumor growth and attenuates muscle wasting in cancer. Cancer Res. 2019;79:706-19.

S47. Boka G, Anglade P, Wallach D, Javoy-Agid F, Agid Y, Hirsch EC. Immunocytochemical analysis of tumor necrosis factor and its receptors in Parkinson's disease. Neurosci Lett. 1994;172:151-4.

S48. Mogi M, Harada M, Riederer P, Narabayashi H, Fujita K, Nagatsu T. Tumor necrosis factor-alpha (TNF-α) increases both in the brain and in the cerebrospinal fluid from parkinsonian patients. Neurosci Lett. 1994;165:208-10.

S49. Samidurai M, Tarale P, Janarthanam C, Estrada CG, Gordon R, Zenitsky G, et al. Tumor Necrosis Factor-Like Weak Inducer of Apoptosis (TWEAK) Enhances Activation of STAT3/NLRC4 Inflammasome Signaling Axis through PKCdelta in Astrocytes: Implications for Parkinson's Disease. Cells. 2020;9:1831.

S50. Johnston AJ, Murphy KT, Jenkinson L, Laine D, Emmrich K, Faou P, et al. Targeting of Fn14 prevents cancer-induced cachexia and prolongs survival. Cell. 2015;162:1365-78.

S51. Tajrishi MM, Sato S, Shin J, Zheng TS, Burkly LC, Kumar A. The TWEAK-Fn14 dyad is involved in age-associated pathological changes in skeletal muscle. Biochem Biophys Res Commun. 2014;446:1219-24.

S52. Mittal A, Bhatnagar S, Kumar A, Lach-Trifilieff E, Wauters S, Li H, et al. The TWEAK-Fn14 system is a critical regulator of denervation-induced skeletal muscle atrophy in mice. J Cell Biol. 2010;188:833-49.

S53. Paul PK, Bhatnagar S, Mishra V, Srivastava S, Darnay BG, Choi Y, et al. The E3 ubiquitin ligase TRAF6 intercedes in starvation-induced skeletal muscle atrophy through multiple mechanisms. Mol Cell Biol. 2012;32:1248-59.

S54. Yadava RS, Foff EP, Yu Q, Gladman JT, Kim YK, Bhatt KS, et al. TWEAK/Fn14, a pathway and novel therapeutic target in myotonic dystrophy. Hum Mol Genet. 2015;24:2035-48.

S55. Hui T, Jing H, Zhou T, Chen P, Liu Z, Dong X, et al. Increasing LRP4 diminishes neuromuscular deficits in a mouse model of Duchenne muscular dystrophy. Hum Mol Genet. 2021;30:1579-90.

S56. Trajanovska S, Ban J, Huang J, Gregorevic P, Morsch M, Allen DG, et al. Muscle specific kinase protects dystrophic *mdx* mouse muscles from eccentric contraction-induced loss of force-producing capacity. J Physiol. 2019;597:4831-50.

S57. Moll J, Barzaghi P, Lin S, Bezakova G, Lochmuller H, Engvall E, et al. An agrin minigene rescues dystrophic symptoms in a mouse model for congenital muscular dystrophy. Nature. 2001;413:302-7.

S58. Ueta R, Sugita S, Minegishi Y, Shimotoyodome A, Ota N, Ogiso N, et al. DOK7 Gene Therapy Enhances Neuromuscular Junction Innervation and Motor Function in Aged Mice. iScience. 2020;23:101385.

S59. Zhao K, Shen C, Li L, Wu H, Xing G, Dong Z, et al. Sarcoglycan Alpha Mitigates Neuromuscular Junction Decline in Aged Mice by Stabilizing LRP4. J Neurosci. 2018;38:8860-73.

S60. Cantor S, Zhang W, Delestree N, Remedio L, Mentis GZ, Burden SJ. Preserving neuromuscular synapses in ALS by stimulating MuSK with a therapeutic agonist antibody. Elife. 2018;7:e34375.

S61. Castellani RJ, Siedlak SL, Perry G, Smith MA. Sequestration of iron by Lewy bodies in Parkinson's disease. Acta Neuropathol. 2000;100:111-4.

S62. Febbraro F, Giorgi M, Caldarola S, Loreni F, Romero-Ramos M. α-Synuclein expression is modulated at the translational level by iron. Neuroreport. 2012;23:576-80.

S63. Dexter DT, Carayon A, Javoy-Agid F, Agid Y, Wells FR, Daniel SE, et al. Alterations in the levels of iron, ferritin and other trace metals in Parkinson's disease and other neurodegenerative diseases affecting the basal ganglia. Brain. 1991;114 ( Pt 4):1953-75.

S64. Lv Z, Jiang H, Xu H, Song N, Xie J. Increased iron levels correlate with the selective nigral dopaminergic neuron degeneration in Parkinson's disease. J Neural Transm (Vienna). 2011;118:361-9.

S65. Reardon TF, Allen DG. Iron injections in mice increase skeletal muscle iron content, induce oxidative stress and reduce exercise performance. Exp Physiol. 2009;94:720-30.

S66. Alves FM, Kysenius K, Caldow MK, Hardee JP, Crouch PJ, Ayton S, et al. Iron accumulation in skeletal muscles of old mice is associated with impaired regeneration after ischaemia-reperfusion damage. J Cachexia Sarcopenia Muscle. 2021;12:476-92.

S67. Halon M, Kaczor JJ, Ziolkowski W, Flis DJ, Borkowska A, Popowska U, et al. Changes in skeletal muscle iron metabolism outpace amyotrophic lateral sclerosis onset in transgenic rats bearing the G93A hmSOD1 gene mutation. Free Radic Res. 2014;48:1363-70.

S68. Alves FM, Kysenius K, Caldow MK, Hardee JP, Chung JD, Trieu J, et al. Iron overload and impaired iron handling contribute to the dystrophic pathology in models of Duchenne muscular dystrophy. J Cachexia Sarcopenia Muscle. 2022;13:1541-53.

S69. Schrag A, Good CD, Miszkiel K, Morris HR, Mathias CJ, Lees AJ, et al. Differentiation of atypical parkinsonian syndromes with routine MRI. Neurology. 2000;54:697-702.

S70. Javanshiri K, Drakenberg T, Haglund M, Englund E. Cardiac Alpha-Synuclein Is Present in Alpha-Synucleinopathies. J Parkinsons Dis. 2022;12:1125-31.

S71. Smith JA, Das A, Ray SK, Banik NL. Role of pro-inflammatory cytokines released from microglia in neurodegenerative diseases. Brain Res Bull. 2012;87:10-20.

S72. Zhou J, Liu B, Liang C, Li Y, Song YH. Cytokine signaling in skeletal muscle wasting. Trends Endocrinol Metab. 2016;27:335-47.

S73. Powers SK, Lynch GS, Murphy KT, Reid MB, Zijdewind I. Disease-Induced Skeletal Muscle Atrophy and Fatigue. Med Sci Sports Exerc. 2016;48:2307-19.

S74. Powers SK, Smuder AJ, Judge AR. Oxidative stress and disuse muscle atrophy: cause or consequence? Curr Opin Clin Nutr Metab Care. 2012;15:240-5.

S75. Kikuchi Y, Shibata M, Hirayanagi K, Nagashima K, Mihara B, Ikeda Y. Putaminal iron deposition precedes MSA-P onset by 2 years. Neurology. 2018;90:1071-2.

S76. Litvan I, Mangone CA, McKee A, Verny M, Parsa A, Jellinger K, et al. Natural history of progressive supranuclear palsy (Steele-Richardson-Olszewski syndrome) and clinical predictors of survival: a clinicopathological study. J Neurol Neurosurg Psychiatry. 1996;60:615-20.

S77. Hoglinger GU, Respondek G, Stamelou M, Kurz C, Josephs KA, Lang AE, et al. Clinical diagnosis of progressive supranuclear palsy: The movement disorder society criteria. Mov Disord. 2017;32:853-64.

S78. Stang CD, Turcano P, Mielke MM, Josephs KA, Bower JH, Ahlskog JE, et al. Incidence and Trends of Progressive Supranuclear Palsy and Corticobasal Syndrome: A Population-Based Study. J Parkinsons Dis. 2020;10:179-84.

S79. Morgan JC, Ye X, Mellor JA, Golden KJ, Zamudio J, Chiodo LA, et al. Disease course and treatment patterns in progressive supranuclear palsy: A real-world study. J Neurol Sci. 2021;421:117293.

S80. Painous C, Marti MJ, Simonet C, Garrido A, Valldeoriola F, Munoz E, et al. Prediagnostic motor and non-motor symptoms in progressive supranuclear palsy: The step-back PSP study. Parkinsonism Relat Disord. 2020;74:67-73.

S81. Kimber J, Mathias CJ, Lees AJ, Bleasdale-Barr K, Chang HS, Churchyard A, et al. Physiological, pharmacological and neurohormonal assessment of autonomic function in progressive supranuclear palsy. Brain. 2000;123 ( Pt 7):1422-30.

S82. Orimo S, Amino T, Itoh Y, Takahashi A, Kojo T, Uchihara T, et al. Cardiac sympathetic denervation precedes neuronal loss in the sympathetic ganglia in Lewy body disease. Acta Neuropathol. 2005;109:583-8.

S83. Albers DS, Beal MF. Mitochondrial dysfunction in progressive supranuclear palsy. Neurochem Int. 2002;40:559-64.

S84. Park LC, Albers DS, Xu H, Lindsay JG, Beal MF, Gibson GE. Mitochondrial impairment in the cerebellum of the patients with progressive supranuclear palsy. J Neurosci Res. 2001;66:1028-34.

S85. Albers DS, Augood SJ, Park LC, Browne SE, Martin DM, Adamson J, et al. Frontal lobe dysfunction in progressive supranuclear palsy: evidence for oxidative stress and mitochondrial impairment. J Neurochem. 2000;74:878-81.

S86. Slade SC, Finkelstein DI, McGinley JL, Morris ME. Exercise and physical activity for people with Progressive Supranuclear Palsy: a systematic review. Clin Rehabil. 2020;34:23-33.

S87 Slade SC, Underwood M, McGinley JL, Morris ME. Exercise and Progressive Supranuclear Palsy: the need for explicit exercise reporting. BMC Neurol. 2019;19:305.

S88. Lee H, Lee MJ, Kim EJ, Huh GY, Lee JH, Cho H. Iron accumulation in the oculomotor nerve of the progressive supranuclear palsy brain. Sci Rep. 2021;11:2950.

S89. Lee SH, Lyoo CH, Ahn SJ, Rinne JO, Lee MS. Brain regional iron contents in progressive supranuclear palsy. Parkinsonism Relat Disord. 2017;45:28-32.

S90. Lai KSP, Liu CS, Rau A, Lanctot KL, Kohler CA, Pakosh M, et al. Peripheral inflammatory markers in Alzheimer's disease: a systematic review and meta-analysis of 175 studies. J Neurol Neurosurg Psychiatry. 2017;88:876-82.
